# Mitochondria-Related Ferroptosis Drives Cognitive Deficits in Neonatal Mice Following Sevoflurane Administration

**DOI:** 10.3389/fmed.2022.887062

**Published:** 2022-07-22

**Authors:** Piao Zhang, Yeru Chen, ShuXia Zhang, Gang Chen

**Affiliations:** Department of Anesthesiology, Sir Run Run Shaw Hospital, School of Medicine, Zhejiang University, Hangzhou, China

**Keywords:** cognitive deficits, mitochondria, ferroptosis, sevoflurane, neonatal

## Abstract

Multiple sevoflurane exposure may result in cognitive deficits in neonatal animals. This study attempted to investigate the potential mechanism of sevoflurane-induced neurotoxicity in developing hippocampus. Neonatal animals received sevoflurane anesthesia, then the behavioral tests and Golgi-Cox staining were employed to detect the effect of sevoflurane inhalation in adult mice. And the mitochondrial function was evaluated using MitoSOX staining, Fluo calcium indicators, mitochondrial permeability transition pore (mPTP) assay, and JC-1 probe after sevoflurane administration. Meanwhile, mitochondrial lipid hydroperoxide and ferroptosis were measured by MitoPeDPP and Mito-FerroGreen signals following sevoflurane exposure. Moreover, the ferroptosis and behavioral performance were assessed after deferiprone (DFP) treatment. The results showed that sevoflurane administration induced cognitive impairment accompanied by reducing dendritic length, density, and nodes. Additionally, sevoflurane exposure elevated mitochondrial ROS production and cytoplasm calcium levels, triggered the opening of mPTP, and decreased the mitochondrial membrane potential (MMP). However, supplement of elamipretide (SS-31) effectively reversed mitochondrial dysfunction. Mitochondrial lipid hydroperoxide production was increased after sevoflurane administration, whereas Fer-1 treatment reduced lipid hydroperoxide formation. Sevoflurane exposure induced mitochondrial iron overload, whereas Mito-Tempo treatment reduced iron accumulation. Prussian blue staining showed that the hippocampal iron deposition was apparently increased after sevoflurane inhalation. Additionally, the ferroptosis-related protein expression (including ACSL4, COX2, GPX4, and FTH1) was significantly changed, whereas DFP effectively suppressed ferroptosis and enhanced sevoflurane-induced behavioral malfunction. These findings demonstrated that sevoflurane administration elicited mitochondrial dysfunction and iron dyshomeostasis and eventually resulted in cognitive impairments, whereas protecting mitochondrial function and chelating neurotoxic iron effectively reversed these pathological processes.

## Introduction

Cognitive dysfunction is one of the most serious complications threatening human health following surgery, which is mainly characterized by learning and memory deficits, visuospatial and executive dysfunctions, and behavioral disorders (1–3). Generally, cognitive deficits would result in the loss of independence, compromised quality of life, and increased mortality, thereby causing a heavy economic and mental burden to the society and families (4). Accumulating evidence revealed that anesthesia may provoke persistent abnormalities of neuronal circuits by affecting neuronal differentiation and synaptogenesis, and pharmacotoxic effects exacerbated the impairment of cognitive domains (5, 6). Anesthesia during childhood may be associated with postoperative behavioral alterations and long-term deficits in neurocognitive development and learning ability (7). Currently, sevoflurane was extensively used in pediatric practice as an inhaled volatile anesthetic agent (8). Previous studies documented that multiple sevoflurane exposure induced neurocognitive impairment in neonatal mice (9). Sevoflurane inhalation aggravated cognitive deficits by initiating neuroinflammation and neurotoxicity in rat hippocampus (10). Sevoflurane administration triggers a series of pathophysiologic reactions comprising mitochondrial dysfunction (11) and endoplasmic reticulum stress (12) and thereby results in cognitive dysfunction by inducing neuronal apoptosis (13), synaptogenesis impairment (14), and neuroinflammation (15).

Mitochondrion, as a cellular organelle, participates in numerous bioenergetic, biosynthetic, and regulatory processes including oxidation-reduction reactions, DNA synthesis and repair, lipid metabolism, and cellular stability (16, 17). Related research suggested that mitochondria were implicated in neural activity, synaptic transmission, neural development, and plasticity (18, 19). Previous studies demonstrated that mitochondrial dysfunction caused a series of diseases including neonatal neurodevelopmental damage (20). More interestingly, mounting evidence demonstrated that the dramatic morphological changes in mitochondria resulted in abnormal absorption and transport of iron and thereby activated a serial cascade of inflammatory responses (21). Of note, iron plays a crucial role in energy metabolism by mediating the electron transport chain, ATP production, and oxygen consumption (22). However, excess iron in the brain altered the behavior and mood (23) and caused learning and memory deficits (24). In this study, we found that cognitive impairment was elicited after multiple neonatal exposures to sevoflurane. Bioinformatic analysis and correlational studies indicated that mitochondria-related ferroptosis may drive cognitive deficits following sevoflurane administration. Our findings revealed that sevoflurane inhalation induced the morphological alteration in mitochondria and iron metabolism abnormality. Whereas, elamipretide (SS-31) effectively improved mitochondrial dysfunction and thereby attenuated cognitive impairment induced by sevoflurane administration. Additionally, ferroptosis accelerated neurological disorders, whereas iron chelator deferiprone (DFP) revered these symptoms. Consequently, this study was conducted to comprehensively illustrate the underlying mechanism concerning sevoflurane-induced neurotoxicity in neonatal mice hippocampus and develop an appropriate therapeutic strategy for attenuating cognitive deficits following sevoflurane administration.

## Materials and Methods

### Animals and Ethical Statement

Neonatal C57BL/6 mice from both sexes at postnatal day 1 were purchased from Shanghai Laboratory Animal Center (Chinese Academy of Sciences, Shanghai, China). The experimental protocols were performed in compliance with the guidelines for laboratory animal care and safety from NIH and approved by the Animal Care and Use Committee of Zhejiang University. All animals were housed in appropriate laboratory conditions (22–25°C, a 12-h light–dark cycle, 45–50% humidity) and supplied tap water and rodent chow *ad libitum*.

### Experiment Grouping and Treatment

All animals were randomly assigned to different groups according to the experimental protocols: Ctrl group, SEV group, SS-31 group, SEV+SS-31 group; Ctrl group, SEV group, DFP group, and SEV+DFP group. To induce general anesthesia, the pups were placed in an acrylic anesthetizing chamber with two interfaces including a sevoflurane vaporizer and a multi-gas monitor. The SEV group was exposed to 3% sevoflurane delivered in humidified 60% O_2_ carrier gas for 2 h daily for 3 days through the Datex-Ohmeda anesthesia system (Madison, WI, USA), whereas the Ctrl group received 60% oxygen (balanced with nitrogen) for the same period at postnatal day 6–8 (P6-8). Similarly, the SS-31 group and DFP group received the same process as the Ctrl group, and the SEV+SS-31 group and SEV+DFP group underwent the same scheme as the SEV group. SS-31 (5 mg/kg, Absin Biotechnology Co., Ltd., Shanghai, China) and DFP (75 mg/kg, Absin Biotechnology Co., Ltd., Shanghai, China) were intraperitoneally administered to the pups at P6 and P8 before SEV inhalation, respectively.

### Behavioral Test

#### Morris Water Maze

After the sevoflurane exposure, the spatial learning and memory abilities were evaluated at P42 using the Morris water maze (MWM) test according to the previously described (25). The swimming track was recorded using a computerized tracking system. In the training phase, all animals received four trials each day for 4 days. In the testing phase, the platform was removed, and the escape latency, platform crossing time, and quadrant time were recorded to assess spatial learning and memory function. After the removal from the pool, animals were manually dried using a terrycloth towel and quickly recovered body temperature *via* a heat lamp for at least 5 min before returning to the home cage.

### Novel Object Recognition Test

Cognition was measured by the novel object recognition (NOR) experiment at P42.

The animals are exposed to two identical objects for 20 min and then trained for 5 min during the familiarization phase. Thereafter, the mice are exposed to a single copy of the familiar object and a novel object (test phase) after 24 h. The total distance traveled was recorded and the recognition index was calculated (26): A recognition index was calculated for each animal and reported as the ratio TB/(TA + TB), where TA = time spent exploring the familiar object A and TB = time spent exploring the novel object B. Recognition memory was evaluated as in the long-term memory test. Exploration was defined as sniffing or touching the object with the nose or forepaws.

### Trace Fear Conditioning

The fear condition test is extensively used to detect the tone's effect on the hippocampus-dependent memory (27). Briefly, the mice were placed in a sound-attenuating fear-conditioning chamber (ACT-100A, Coulbourn Instruments, USA). The mice freely explored the chamber for 2 min, and the freezing was recorded as control. Then, the mice received a 30-s sound (80 dB, 1,500 HZ) as a conditioned stimulus and foot shock (0.7 mA; 2 s) *via* the floor's steel rods at the last 2 s, and keep the sound and the shock stopped at the same time. The mice stayed in the chamber for another 2 min. The training was repeated 5 times. The next day for the contextual fear test, the mice were placed into the same chamber and the freezing was recorded for 5 min. After 2 h, the mice were placed into another chamber for 3 min and then received the same conditioned stimulus for 3 min. The freezing of mice was recorded all the time. The data of freezing were recorded by Freeze Frame software.

### Immunofluorescent Staining

Animals were sacrificed with excessive 1% pentobarbital sodium. Brain tissues were acquired and fixed in 4% paraformaldehyde at 4°C overnight and then sequentially dehydrated with 10, 20, and 30% sucrose solution. Serial coronal slices (20 μm) were made using a rotary microtome (Leica, Germany). The sections were placed in a water bath (96°C, 20 min) for antigen retrieval and blocked through 10% bovine serum albumin (room temperature, 1 h). The sections were incubated with antibody diluent containing goat antibodies against Tuj1 (1:500; Biolegend, A488-435L), GFAP (1:100; ABclonal, A14673), and NeuN (1:1000; Abcam, ab104224) overnight at 4°C. Then, sections were rinsed with PBS followed by incubation with Alexa Fluor™ 488 goat anti-mouse antibody and Alexa Fluor™ 594 goat anti-rabbit antibody for 1 h at room temperature. Fluorescence signals were visualized by an epifluorescence microscope, and images were captured through ImagePro Plus 5.0 software.

### Golgi-Cox Staining

Golgi-Cox staining was performed to investigate the morphology of neuronal dendrites and dendritic spines using the Hito Golgi-Cox OptimStain™ PreKit (Hitobiotec Corp. Kingsport, TN, USA). The brain tissues were obtained and rinsed with Milli-Q water. The brain tissues were impregnated by the equal volumes of Solutions A and B, and then, the impregnation solution was replaced the following day and stored in darkness for 2 weeks at room temperature. The brain tissues were transferred to Solution C and stored for 72 h (4°C). The sections (100 μm) were generated using a cryotome. A drop of Solution C was placed on gelatin-coated microscope slides. The remaining Solution C was wiped away through a strip of filter paper and allowed to dry naturally for 3 days. The dried slices were stained following the manufacturer's manual. Thereafter, morphological analyses (CA1 region) were observed using an Olympus BX61 fluorescence microscope (Olympus, Japan).

### High-Throughput RNA Sequencing

Total RNAs were extracted from the hippocampal tissues using RNAiso Plus Reagent (TaKaRa, Japan), and purified through RNasey Mini Kit (QIAGEN). The total RNAs were identified and quantified by NanoDrop spectrophotometry (Thermo Scientific, Wilmington, USA), and 1–2 μg of total RNAs was used to construct the RNA-Seq libraries according to the manufacturer's protocol. Sequencing library was detected using an Illumina HiSeq platform, and paired-end reads were generated followed by cluster generation. The raw sequencing data were processed by in-house Perl scripts after quality control. Thereafter, the differentially expressed genes (FDR < 0.05, fold change >2) were analyzed using R studio software. Gene Ontology (GO) analysis and Kyoto Encyclopedia of Genes and Genomes (KEGG) pathway were performed using String online tools (https://string-db.org). Then, the RNA-seq data files were deposited in the NCBI Sequence Read Archive (SRA) database (SRA accession: PRJNA781082).

### Cell Culture

Primary hippocampal neurons were cultured according to the previously described (28). Briefly, the fetal hippocampi (E17) were obtained under a sterile environment, then treated with 0.125% trypsin in Hank's buffer (in mmol/L: 137 NaCl, 5.4 KCl, 0.4 KH2PO4, 0.34 Na2PO4·7H2O, 10% glucose, and 10 HEPES) for 12 min at 37°C, and dissociated through repeated passage with Pasteur pipettes. The nerve cells (2 × 10^5^/cm^2^) were seeded onto poly-l-lysine (10 μg/mL)-coated plates with neurobasal medium (Invitrogen), 2% B27 (Invitrogen), 10 U/ml penicillin, 10 U/ml streptomycin, and 0.5 mmol/L glutamine. The cells were continuously cultured for 20 days and then harvested for subsequent experiments. Meanwhile, H4 human neuroglioma was obtained from the China Center for Type Culture Collection. These cells were cultured using Dulbecco's Modified Eagle's Medium (DMEM) supplemented with 10% F12 (all from Gibco, Grand Island, NY, USA) and 10% heat-inactivated fetal bovine serum in a humidified incubator (37°C, 5% CO_2_).

### Sevoflurane Treatment

The cultured cells were placed in an airtight plastic chamber (MIC-101), which was connected to an acrylic anesthetizing chamber with two interfaces including a sevoflurane vaporizer and a multi-gas monitor. The chamber was gassed with 4.1% sevoflurane in the carrier gas (95% air/5% CO_2_) for 15 min, and the concentration of sevoflurane was monitored by a gas monitor (PM 8060, Drager, Lübeck, Germany). Then, the chamber was sealed and incubated for 6 h at 37°C. The gas was renewed every 3 h, and the concentration of sevoflurane was determined. Meanwhile, the control group received the same process with air containing 5% CO_2_.

### Measurement for Mitochondrial Function

Mitochondrial function was detected including ROS production, mitochondrial permeability transition pore (mPTP), calcium loading, and mitochondrial membrane potential (MMP). Mitochondrial ROS accumulation was detected by the MitoSOX reagent (M36008, Thermo Fisher, USA). Meanwhile, the ROS level was measured when using a DMEM medium without glutamine, supplied with α-KG (2, 4, 8, and Fer-1+8 mM; Sigma, 75890), rotenone (10 μm; MCE, HY-B1756) or antimycin A (50 μm; MCE, HY-107406), respectively. Mitochondrial lipid peroxidation and iron content were detected by MitoPeDPP (Dojindo, M466) and Mito-FerroGreen (Dojindo, M489) after adding Mito-Tempo (10 μm; MCE, HY-112879) or Fer-1 (1 μm; MCE, HY-100579). The Mitochondrial Permeability Transition Pore Assay Kit (C2009S, Beyotime, China) and the JC-1 mitochondrial membrane potential assay kit (Thermo Fisher Scientific, MA, USA) were used to determine mitochondrial damage. Calcium mobilization was detected by Fluo calcium indicators (Fluo-4, AM, YEASEN, 40704ES50). The level of lipid peroxidation was detected by the fluorescent reporter molecule C11-BODIPY^581/591^ (Invitrogen^TM^, D3861). Additionally, the morphology of mitochondria in the hippocampal neurons was observed using a transmission electron microscope (TEM) (Philips Tecnai 10, Holland) in the Center of Cryo-Electron Microscopy at Zhejiang University as described previously (29). All experimental protocols described above were performed following the manufacturer's instructions.

### Seahorse Measurement

The oxygen consumption rate (OCR) was measured using a Seahorse XF96 analyzer (Seahorse Agilent, USA) combined with the Agilent Seahorse XFe96 Extracellular Flux Assay Kit according to the manufacturer's recommendations. Briefly, cells were treated with 8 μm oligomycin, 9 μm carbonyl cyanide 4-(trifluoromethoxy) phenylhydrazone (FCCP), 20 μm rotenone, and 100 μm antimycin A as described previously (30).

### Determination of ATP, Malondialdehyde (MDA), and GSH Levels

The ATP concentration was quantified by a fluorometric assay kit (Beyotime, S0026). The MDA concentration was evaluated using the Lipid Peroxidation MDA Assay Kit (Beyotime, S0131). Additionally, the level of GSH was measured according to the requirements of the instructions in the reagent kits (Beyotime, S0052).

### Iron Level Detection

Iron assay was performed according to the manufacturer's protocol of the Iron Assay Kit (Abcam, ab83366) (31). Briefly, the specimens were incubated with an iron reducer at 25°C for 30 min followed by incubating for 60 min with an iron probe at 25°C. Then, the microplate reader (OD 593 nm) was used to detect the level of iron.

### Prussian Blue Staining

Sections (5 μm) were stained for Prussian blue reaction through an Iron Stain Kit (YEASEN, 60533ES20) according to the manufacturer's instructions. Briefly, slides were deparaffinized and hydrated to deionized water. Then, the samples were immersed in a freshly prepared solution of equal parts 5% potassium ferrocyanide and 5% hydrochloric acid for 10 min. The samples were rinsed using deionized water, immersed in 2% pararosaniline solution for 5 min, rinsed with deionized water once again, and immediately dehydrated and coverslipped. Images of positively stained sections were captured *via* an Olympus BX61 microscope.

### Western Blot

The hippocampal tissues, primary cultured neurons, and cell lines were homogenized using RIPA buffer (Beyotime, P0013B) with a 1 × protease inhibitor cocktail (Beyotime, P1010). The supernatant was collected by centrifugation (16, 200 × *g*, 10 min), and the protein concentration was measured through a bicinchoninic acid protein assay kit (Beyotime, P0012S). An aliquot of 50 μg protein was separated *via* SDS-PAGE, transferred to a nitrocellulose membrane, and then blocked with 5% nonfat milk in phosphate-buffered saline (PBS, pH 7.4). The membranes were incubated with primary antibodies against ACSL4 (1:500; ABclonal, A16848), FTH1 (1:500; ABclonal, A19544), GPX4 (1:1, 000; Abcam, ab125066), COX2 (1:1,000; ABclonal, A1253), and actin (1:5, 000; ABclonal, AC026) at 4°C overnight. Blots were incubated in horseradish peroxidase-conjugated secondary antibodies against rabbit IgG (1:5, 000, CST, 7071 and 7072) for 2 h at room temperature, then subjected to chemiluminescent detection using the SuperSignal West Pico Substrate (34077, Pierce), and exposed to film. Digital images were quantified using densitometric measurements obtained using Quantity One software (Bio-Rad).

### Statistical Analysis

GraphPad Prism 8 was used to process the data. All data were represented as mean ± SD. The unpaired *t-*test was used when comparing both sets of data. The one-way ANOVA was performed for univariate ANOVA of an independent sample. For the one-way ANOVA, we performed a Tukey's test for *post-hoc* comparison. Additionally, the two-way ANOVA was employed when there were two factors and they were independent. For two-way ANOVA, we performed a Bonferroni test for *post-hoc* comparison. *p-v*alue of < 0.05 was considered statistically significant.

## Results

### Sevoflurane Exposure Induced Cognitive Impairment in Developing Mice Implicated in Neuronal Death and Abnormal Synapse Formation

**Figure 1:**
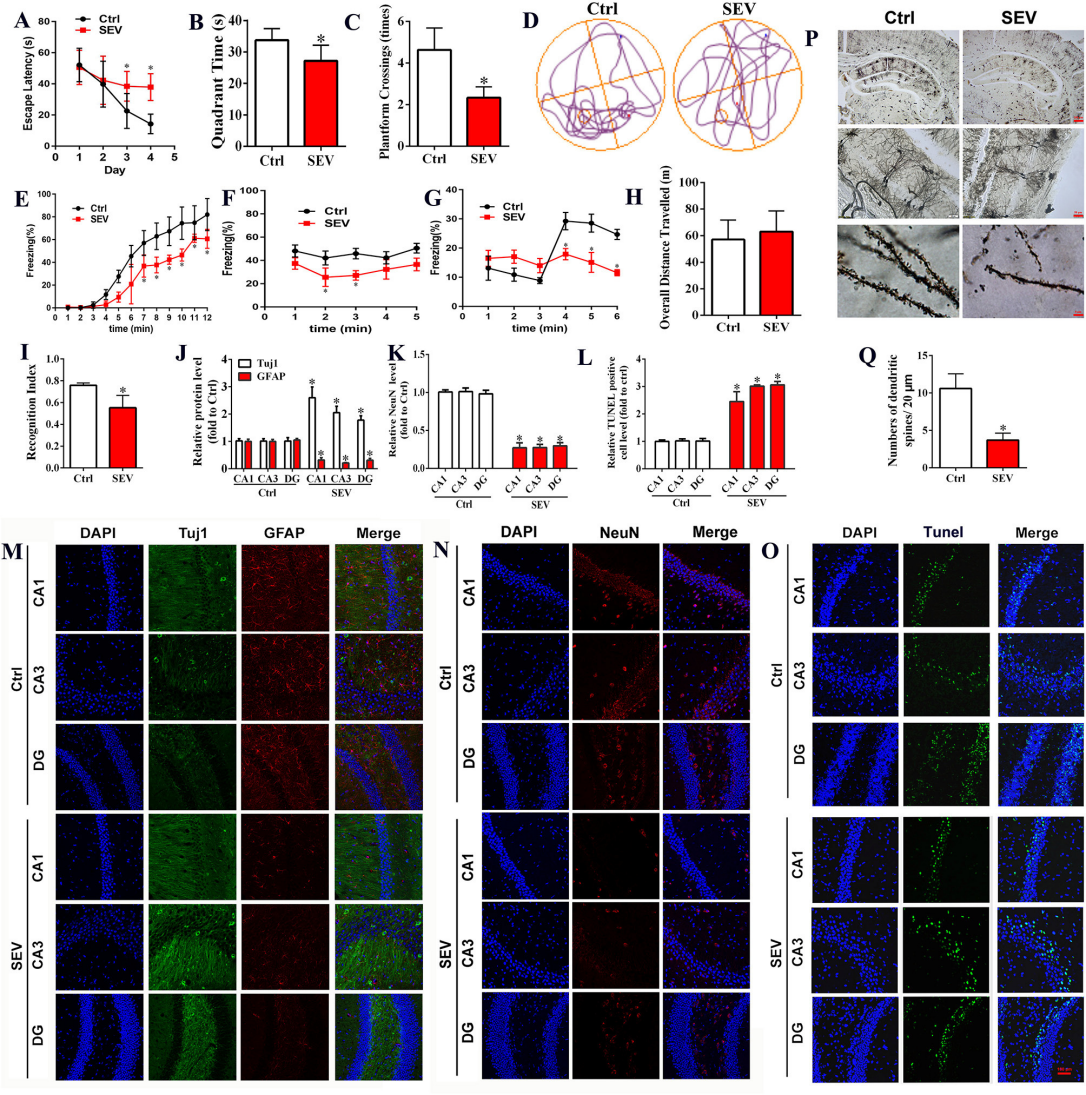
Sevoflurane exposures induced cognitive impairment in neonatal mice associated with neuronal death and abnormal synapse formation. **(A–D)** Morris water maze tests including the time course of escape latency, the time spent in the target quadrant, platform crossing number, and moving track [**(A)**: two-way ANOVA, Bonferroni's multiple comparisons test, interaction: *F*_(3,60)_ = 7.34, mean difference (95% CI): −15.14 (−26.38 to −3.897) (Day 3 Ctrl vs. SEV) and −23.99 (−35.23 to −12.75) (Day 4 Ctrl vs. SEV), *p* < 0.001; **(B)**: *t-*test, *p* = 0.0097; **(C)**: *t-*test, *p* < 0.001; *n* = 8]; **(E–G)** freezing behavior detected by fear condition test, including the percentage of freezing when mice received training [**(E)**: two-way ANOVA, Bonferroni's multiple comparisons test, interaction: *F*_(11,60)_ = 2.590, mean difference (95% CI): 16.25 (−0.04037 to 32.54)(7 min), 20.93 (4.643 to 37.22) (8 min), 19.58 (3.288 to 35.87) (9 min), 22.54 (6.248 to 38.83) (10 min), 7.632 (−8.657 to 23.92) (11 min), 17.42 (1.133 to 33.71) (12 min), *p* < 0.01], without sound stimulus and foot shock [**(F)** two-way ANOVA, Bonferroni's multiple comparisons test, interaction: *F*_(4,25)_ = 0.1686, mean difference (95% CI): 15.27 (−15.13 to 45.67) (2 min) and 18.47 (−11.92 to 48.87) (3 min), *p* < 0.05], and with only sound stimulus [**(G)** two-way ANOVA, Bonferroni's multiple comparisons test, interaction: *F*_(5,30)_ = 3.463, mean difference (95% CI): 9.258 (−4.760 to 23.28) (4 min), 13.21 (−0.8051 to 27.23) (5 min), and 12.32 (−1.697 to 26.34) (6 min), *p* < 0.05; *n* = 6**)**; **(H,I)** novel object recognition comprising of the overall distance of traveling and the recognition index [**(H)**: *t-*test, *p* > 0.05; **(I)**: *t-*test, *p* < 0.001; *n* = 8); **(J,K,M,N)** immunofluorescent staining for Tuj1, GFAP, and NeuN in hippocampus [**(J,K)**: *t-*test, *p* < 0.05); **(L,O)** cell apoptosis detection by TUNEL staining (*t-*test, *p* < 0.05); **(P,Q)** Golgi-Cox staining for analyzing synapse formation (*t-*test, *p* < 0.001). Scar bar = 100 μm, applied in **(M–O)**. *p < 0.05, Ctrl vs. SEV.

To determine the effect of sevoflurane administration on cognitive function, behavioral tests including MWM, novel object recognition test, and trace fear conditioning were employed. The results showed that sevoflurane inhalation in neonatal mice induced cognitive impairment as measured by MWM tests including escape latency, target quadrant time, platform crossing number, and motion trail when compared to the Ctrl group (Figures 1A–D, *p* < 0.05). The fear condition test showed that the freezing time was reduced in the SEV group than that of the Ctrl group (Figures 1E–G, *p* < 0.05). The novel object recognition test suggested that the overall distance of traveling was not significantly different (Figure 1H, *p* > 0.05), whereas the recognition index was decreased in the SEV group compared to the Ctrl group (Figure 1I, *p* < 0.05). The immunofluorescence staining results showed that the Tuj1-positive cells were obviously increased, whereas the NeuN and GFAP-positive cells were decreased in the SEV group consisting of CA1, CA3, and DG regions compared to the Ctrl group (Figures 1J,K,M,N; *p* < 0.05). The TUNEL assay showed that the apoptosis rate was increased in the SEV group including CA1, CA3, and DG regions than that of the Ctrl group (Figures 1L,O; *p* < 0.05). Meanwhile, the results displayed that the dendritic length, density, and nodes were significantly reduced after sevoflurane administration compared with the Ctrl group indicated by Golgi-Cox staining (Figures 1P,Q; *p* < 0.05). These results demonstrated that sevoflurane anesthesia resulted in neuronal death and abnormal synapse formation and induced cognitive dysfunction in neonatal animals.

### Bioinformatic Analysis for RNA-seq Data

**Figure 2:**
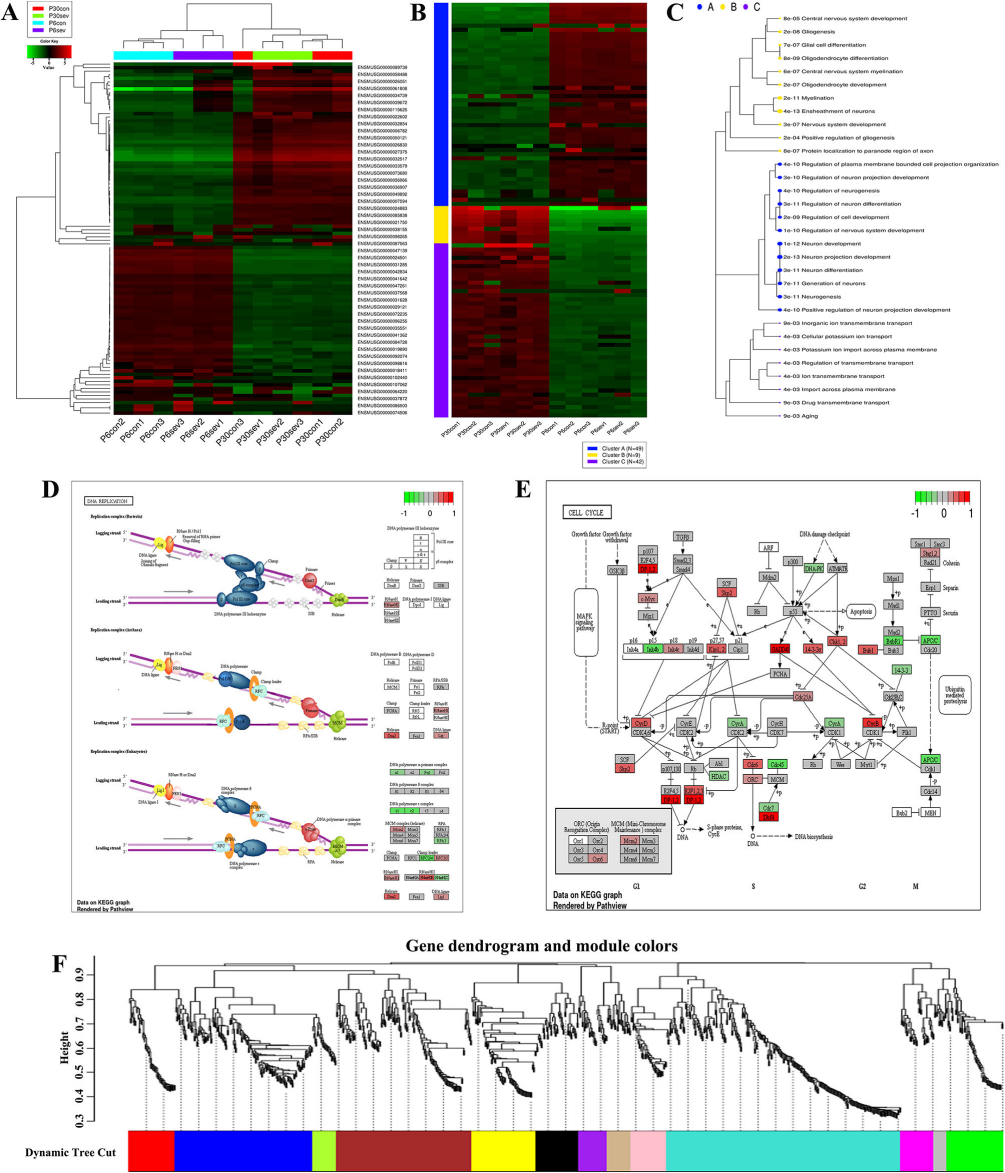
Bioinformatic analysis for RNA-Seq data. **(A)** Hierarchical clustering of top 100 genes, and the color scale represents the relative genes expression in certain slide: green indicates low relative expression levels, red indicates high relative expression levels, and black indicates no change; **(B)** K-means clustering; **(C)** Enrichment analysis for GO terms, sizes of dot correspond to adjusted *p*-values; **(D,E)** parametric analysis of gene set enrichment (PGSEA) for the potential interaction networks and signaling pathways; **(F)** weighted gene correlation network analysis (WGCNA) for investigating the underlying correlations among genes and groups using network topology.

To further clarify the potential mechanism of sevoflurane-induced neurotoxicity, RNA-seq was performed and the data were analyzed using R studio software. First, the gene ID was converted and filtered. Then, all genes were ranked based on the standard deviation, and the hierarchical clustering was drawn across all samples using the top 100 genes (Table 1). The graph showed that sevoflurane administration contributed to significant changes in gene expression (Figure 2A). The top 100 genes were divided into four groups using k-means clustering (Figure 2B), and the enriched GO terms are shown in Figure 2C (data details: Supplementary Tables S1, S2). Thereafter, parametric analysis of gene set enrichment (PGSEA) was performed, and Figures 2D,E separately showed the potential interaction networks and signaling pathways involved in DNA replication and cell cycle. Moreover, weighted gene correlation network analysis (WGCNA) was employed to investigate the potential correlations among genes and groups using network topology, and the results showed that these genes were clearly delineated in 13 modules (Figure 2F). The results suggested that sevoflurane administration triggered a series of gene expression changes, which are implicated in the nervous system development including neuron differentiation and projection, and neurogenesis.

**Table 1:** The hierarchical clustering was drawn by using the top 100 genes.

		**P6con2**	**P6con1**	**P6con3**	**P6sev3**	**P6sev2**	**P6sev1**	**P30con3**	**P30sev1**	**P30sev2**	**P30sev3**	**P30con1**	**P30con2**
ENSMUSG00000099227	Mir8114	0.339139	0.639115	0.773078	0.813188	0.727697	1.057615	8.513753	8.709748	8.764851	0.809721	0.52445	0.516952
ENSMUSG00000089739	Gm20431	0	0	0	0	0	0	0	5.512488	0	0	0	0
ENSMUSG00000102543	Pcdhgc5	1.540981	2.474268	2.981063	2.53654	5.313874	1.821173	3.22469	5.200865	5.106705	3.613448	5.326615	3.009621
ENSMUSG00000058488	Kl	0.643611	0.679354	0.855867	0.404465	1.337473	1.608458	1.81228	4.231118	3.980575	3.774451	3.603163	4.577633
ENSMUSG00000022425	Enpp2	2.596566	2.69461	3.155094	2.854968	5.003974	5.329215	4.483309	8.162468	7.870834	7.761079	7.505069	8.720711
ENSMUSG00000026051	1500015O10Rik	2.320001	2.721176	2.426835	2.669784	4.739422	5.290961	1.488714	5.427677	5.423005	5.423128	5.275562	6.368469
ENSMUSG00000004655	Aqp1	0.557267	0.602999	0.603644	0.691891	3.472448	3.937689	0.281892	3.454538	3.451919	3.13121	2.872202	3.648984
ENSMUSG00000061808	Ttr	0.60029	2.088285	2.793013	1.957387	10.64071	11.09667	4.813186	11.57848	11.44686	11.29509	11.24899	12.17576
ENSMUSG00000079436	Kcnj13	0.052239	0.281671	0.603794	0.085789	2.065224	2.645705	0.252962	4.297776	4.290035	4.059775	3.860188	4.782417
ENSMUSG00000034739	Mfrp	0.037954	0.082597	0.083571	0.097811	1.396841	1.97803	0.280518	3.235523	3.351814	2.84732	2.670659	3.463689
ENSMUSG00000037086	Prr32	0	0	0.170719	0	2.314767	2.530235	0.178631	2.985613	3.039295	2.870868	2.745784	3.324548
ENSMUSG00000039672	Kcne2	0	0	0	0.062171	2.761808	3.117162	0.185855	3.768732	3.251112	3.404531	3.285776	4.341999
ENSMUSG00000022949	Clic6	0.207005	0.348554	0.365233	0.282579	3.168213	3.282571	0.880122	4.438601	4.341677	4.100857	3.937147	4.816006
ENSMUSG00000115625	2900040C04Rik	0	0	0	0	2.433959	2.891823	0.436016	3.821567	3.692222	3.721285	3.621763	4.458132
ENSMUSG00000046093	Hpcal4	2.890394	4.03528	3.389683	2.743864	3.534995	3.096736	5.357839	7.632003	5.30247	7.505601	4.420493	5.321748
ENSMUSG00000022602	Arc	2.186194	2.283747	2.383563	2.002078	2.484504	2.487388	5.042014	6.054658	6.572029	6.428091	4.782327	4.650254
ENSMUSG00000030701	Plekhb1	2.762791	2.762292	2.826443	2.754226	2.562928	2.613716	5.580811	4.832645	6.071605	5.212776	5.109616	5.308182
ENSMUSG00000032854	Ugt8a	1.048664	0.952906	1.271944	1.073586	1.162132	1.302902	4.123735	3.504565	4.221014	4.145976	4.533896	4.726676
ENSMUSG00000043448	Gjc2	0.389721	0.165084	0.372247	0.186042	0.550101	0.368633	3.587328	2.714377	3.472717	3.551479	4.007932	3.977796
ENSMUSG00000006782	Cnp	3.912011	3.800569	3.899937	3.656499	3.807092	4.141042	6.805457	6.017177	7.072083	7.001268	7.230329	7.253384
ENSMUSG00000032554	Trf	2.318652	2.225575	2.678687	2.283432	2.362017	2.655249	5.853685	5.619558	6.399286	6.210752	6.295796	6.435897
ENSMUSG00000050121	Opalin	0	0	0.053712	0.117255	0	0	4.041932	3.248058	4.427679	4.237906	4.389326	4.329293
ENSMUSG00000036634	Mag	0.438031	0.390555	0.38147	0.407697	0.495347	0.712602	5.21762	4.539451	5.638096	5.626706	5.746751	5.734548
ENSMUSG00000026830	Ermn	0.209977	0.280025	0.297944	0.316506	0.32334	0.267793	3.709827	3.456199	4.314028	4.307012	4.261882	4.471121
ENSMUSG00000037625	Cldn11	1.717406	2.168798	2.296076	1.855062	2.343677	2.043547	6.850832	6.071192	7.208068	7.131991	7.27962	7.363363
ENSMUSG00000027375	Mal	0.468541	1.056645	1.177564	0.696692	1.061306	1.060291	6.881021	6.121725	7.208538	7.161206	7.252946	7.406027
ENSMUSG00000031425	Plp1	1.184008	1.381746	1.535831	1.082337	1.55125	1.689505	8.046047	7.371442	8.510116	8.353888	8.502598	8.780668
ENSMUSG00000032517	Mobp	0.176754	0.126103	0.071435	0.032413	0.078641	0.091435	7.079269	6.60952	7.701476	7.530721	7.393925	7.725572
ENSMUSG00000041607	Mbp	2.900006	2.975117	2.868769	2.486042	2.879045	2.897536	7.021765	6.755859	7.578742	7.369391	7.204559	7.468361
ENSMUSG00000033579	Fa2h	0.721697	0.523699	0.760457	0.467907	0.371569	0.713993	4.426835	3.586859	4.512877	4.342097	4.368211	4.870577
ENSMUSG00000076439	Mog	0.949917	0.757949	1.010251	0.798649	0.592399	0.676379	5.655879	4.790305	5.873042	5.837576	6.13841	6.235318
ENSMUSG00000073680	Tmem88b	0.66543	0.483404	0.364741	0.317987	0.232424	0.106453	4.127202	3.527263	4.328289	4.472316	4.378304	4.617043
ENSMUSG00000037166	Ppp1r14a	0.655845	0.84407	0.647737	0.826795	0.506365	0.534378	3.589188	2.91349	3.879721	3.621852	3.772591	4.005399
ENSMUSG00000056966	Gjc3	0.424219	0.551056	0.374767	0.340942	0.41214	0.552782	3.297991	2.727668	3.688249	3.565231	3.377548	3.765232
ENSMUSG00000026879	Gsn	2.225278	2.557166	2.65014	2.231915	2.991605	3.126786	5.20788	4.665979	5.450108	5.332465	5.677892	5.814907
ENSMUSG00000036907	C1ql2	1.603376	1.535475	2.055736	2.262293	2.178253	2.094157	5.313709	5.180862	5.075851	5.107971	5.130215	4.856868
ENSMUSG00000107272	Gm34583	0	0	0.194649	0	0.335162	0	3.297843	2.669013	3.473341	3.491618	3.013496	3.170002
ENSMUSG00000049892	Rasd1	1.415436	1.851232	1.804416	1.537303	1.824477	1.67218	4.95257	4.430186	4.950103	4.796765	4.6052	4.392877
ENSMUSG00000005716	Pvalb	0.40242	0.37835	0.260647	0.200058	0.285336	0.687405	4.486644	4.438122	4.477046	4.589179	4.680512	4.279853
ENSMUSG00000007594	Hapln4	0.67364	0.768904	0.771197	0.680055	0.711176	0.786114	5.114192	4.949516	4.952833	4.841899	4.878249	4.671705
ENSMUSG00000074968	Ano3	0.877627	0.891124	1.00526	0.744247	0.639223	1.028931	3.937192	3.916454	4.035102	3.854804	3.722682	3.8312
ENSMUSG00000024883	Rin1	1.182804	1.628938	1.53163	1.128684	1.384394	1.225824	4.191679	4.176614	4.029093	4.186772	3.992423	4.046182
ENSMUSG00000017167	Cntnap1	1.859463	2.379732	2.362483	1.866618	2.015137	2.401964	5.294768	5.342973	5.29708	5.226029	5.1251	5.06464
ENSMUSG00000085838	Chn1os1	4.463286	4.940295	5.015282	4.336657	4.744263	5.035754	8.203587	8.017724	7.764591	7.755341	7.726485	7.657788
ENSMUSG00000064353	mt-Td	1.419549	1.652578	1.841132	1.28329	1.704404	1.592377	4.271732	4.43539	4.680096	4.258663	3.934356	4.333975
ENSMUSG00000021750	Fam107a	3.323282	3.500449	3.761516	3.131521	3.763401	3.752174	6.484659	6.747674	6.748346	6.655248	6.354992	6.557933
ENSMUSG00000104797	Mir1897	0.018215	3.752156	2.296082	0.092475	0.0276	3.072096	3.894131	0.048836	3.089385	0.309142	0.002138	4.190689
ENSMUSG00000038155	Gstp2	0	2.78686	2.176886	0	0	2.740684	0	2.687933	2.884693	3.460866	0	1.195596
ENSMUSG00000065968	Ifitm7	0.034546	3.406845	3.418375	3.253375	0.119296	3.299889	2.968475	3.059169	2.983583	2.949681	2.724876	0.056279
ENSMUSG00000098265	Gm27517	0.372889	0.41968	0.327247	0.147016	5.229758	0.405011	4.488657	0.138088	0.052523	0.038445	0.402865	4.467028
ENSMUSG00000116048	2-Sep	1.647756	1.375676	4.653767	2.182891	4.639728	1.420056	3.911606	1.504378	0	3.988841	3.687734	4.172691
ENSMUSG00000087563	Gm14205	0	0	4.722188	0	0	0	0	0	0.013082	0	4.450827	0
ENSMUSG00000029168	Dpysl5	5.956252	5.933384	5.961096	5.957929	5.76294	5.962918	2.675896	2.99459	2.806494	3.073291	2.835077	2.980861
ENSMUSG00000047139	Cd24a	7.412661	7.164381	7.280045	7.544802	7.230405	7.235592	2.784359	3.135097	2.85593	3.070662	3.025006	3.284487
ENSMUSG00000022865	Cxadr	6.127147	6.181832	6.293292	6.225999	6.057464	6.149617	2.010012	1.966	2.045834	1.846655	1.863141	2.140173
ENSMUSG00000024501	Dpysl3	8.574621	8.591108	8.61324	8.653219	8.459495	8.583664	3.610343	3.751818	3.460116	3.649018	3.610066	3.734979
ENSMUSG00000036913	Trim67	5.305222	5.265061	5.265873	5.503747	5.146142	5.36841	0.877609	1.021301	1.14274	1.138241	1.072901	0.960284
ENSMUSG00000031285	Dcx	5.27977	5.219535	5.420898	5.483797	5.111164	5.345898	1.24005	1.328293	1.332337	1.354823	1.26626	1.366695
ENSMUSG00000025789	St8sia2	5.12088	5.009673	4.795467	5.205127	4.839677	4.733361	0.903026	0.888194	0.743614	0.801173	0.749059	0.846253
ENSMUSG00000042834	Nrep	8.730464	8.694268	8.510802	8.686963	8.480076	8.464634	4.493812	4.585504	4.589183	4.485819	4.538477	4.622351
ENSMUSG00000074480	Mex3a	3.615988	3.591072	3.571556	3.834256	3.452504	3.630161	0.938506	0.745443	0.968905	0.937242	0.86646	0.920821
ENSMUSG00000041642	Kif21b	5.492827	5.688199	5.635442	5.587384	5.451578	5.637688	2.645654	2.879272	2.71721	2.750449	2.607779	2.650084
ENSMUSG00000018012	Rac3	7.198696	6.963205	6.959161	7.074956	6.980815	6.90353	3.436679	3.661962	3.165043	3.42661	3.678316	3.36163
ENSMUSG00000047261	Gap43	9.281286	9.18918	9.199478	9.367284	9.213413	9.134252	6.528261	6.702916	6.29199	6.559983	6.649484	6.631088
ENSMUSG00000079523	Tmsb10	9.056489	8.834222	8.712459	8.807929	8.646697	8.56338	5.896677	5.940514	5.900179	6.119379	5.94729	5.958088
ENSMUSG00000037568	Vash2	3.476223	3.339786	3.230416	3.698707	3.432289	3.426754	0.847694	0.437191	0.642145	0.773907	0.659888	0.59595
ENSMUSG00000029005	Draxin	4.08241	3.857957	3.931712	4.17607	3.686309	3.95187	0.735826	0.642166	0.559072	0.487562	0.767514	0.658067
ENSMUSG00000031628	Casp3	5.31873	5.108197	5.123518	5.401578	5.048968	5.114293	2.157852	1.817973	2.008147	1.725975	2.058693	2.044331
ENSMUSG00000045136	Tubb2b	10.4977	10.4071	10.33577	10.52977	10.35218	10.41949	7.463822	7.04191	7.20682	7.274981	7.438784	7.212073
ENSMUSG00000029121	Crmp1	7.923535	7.852199	7.930473	7.994561	7.865559	7.927148	5.371702	5.08746	5.135819	5.075436	5.279559	5.055205
ENSMUSG00000047945	Marcksl1	9.473849	9.287881	9.230921	9.410577	9.272566	9.152915	6.502186	6.328559	6.409094	6.34537	6.691804	6.343286
ENSMUSG00000072235	Tuba1a	11.72084	11.63042	11.56435	11.76712	11.66514	11.58274	9.002722	8.816189	8.893305	8.972796	9.165928	8.910132
ENSMUSG00000044847	Lsm11	5.691031	5.901785	6.011299	5.744274	5.816376	5.985011	3.362233	3.115294	3.137247	3.112455	3.209757	3.112524
ENSMUSG00000096255	Dynlt1b	6.363808	6.243093	6.292348	6.132596	6.285557	6.267778	3.589882	3.20411	3.475759	3.598	3.791634	3.693685
ENSMUSG00000070527	Mkrn3	3.423404	3.235095	3.412129	3.313038	3.186531	3.371228	0.583462	0.436844	0.605046	0.77883	0.943553	0.537681
ENSMUSG00000035551	Igfbpl1	5.095995	4.890607	5.16656	5.284854	4.86535	5.277536	2.261028	1.716814	2.052984	1.709055	2.045768	2.293702
ENSMUSG00000028364	Tnc	4.211886	4.160709	4.343726	4.271429	3.806884	4.173232	1.254142	0.92249	1.181049	1.078118	1.270066	1.309538
ENSMUSG00000041362	Shtn1	5.782274	5.769358	5.777735	6.005109	5.703314	5.74011	2.716395	2.685376	2.927242	2.958568	3.112713	3.365067
ENSMUSG00000044338	Aplnr	3.048	3.168751	3.402846	3.253904	3.163615	3.067377	0.186222	0.129845	0.034183	0.244871	0.634228	0.474575
ENSMUSG00000084728	Mir1905	4.404927	4.201791	4.298829	4.622215	4.106126	4.476413	1.021752	0.877809	1.598562	0.804981	0.739724	1.117915
ENSMUSG00000019874	Fabp7	8.114886	7.887725	8.028364	8.086709	7.677698	7.902722	2.969595	3.79241	4.145775	4.024288	3.590086	3.681293
ENSMUSG00000019890	Nts	5.880063	5.780624	5.638106	6.00656	5.568164	5.497426	2.142706	2.470744	2.514862	3.018826	1.896446	2.262631
ENSMUSG00000095677	Dynlt1f	4.64278	4.261188	4.603206	4.666007	4.444555	4.49313	1.724146	1.497589	1.25608	1.90696	2.31641	1.813998
ENSMUSG00000092074	Dynlt1a	5.376143	5.163592	5.238181	5.043642	5.111786	5.194224	1.448215	1.250842	1.768933	1.357393	2.418522	1.98848
ENSMUSG00000094392	Gm3788	5.263658	4.863463	4.979568	4.841488	5.130374	4.362327	2.286998	2.711215	1.513535	2.18349	2.057185	1.918746
ENSMUSG00000098816	Gm27786	1.807631	3.763076	3.408871	3.15314	3.441816	3.592642	0.202129	0.501566	0.409775	0	0.066467	0.416267
ENSMUSG00000105211	Gm47302	1.789459	3.449842	4.341674	3.166937	3.418886	3.164747	1.347937	0.084804	1.236344	0.078889	0.766359	1.299217
ENSMUSG00000018411	Mapt	7.965571	5.914616	6.009106	5.931301	7.81837	6.0846	4.028522	4.32592	4.196329	5.332961	3.980954	3.902854
ENSMUSG00000023036	Pcdhgc4	3.034378	3.043546	4.998936	6.340767	4.823971	5.837487	4.161959	3.113443	1.636678	1.571729	1.784668	4.086564
ENSMUSG00000102440	Pcdhga9	6.346165	1.824466	1.422592	2.573931	3.442794	1.399153	1.289931	1.057458	1.66033	1.310436	0.989857	2.32774
ENSMUSG00000103585	Pcdhgb4	1.621667	5.718772	5.144098	1.272979	1.102232	1.479735	1.488937	1.116522	1.125121	1.002364	0.89602	1.395783
ENSMUSG00000107062	Gm43580	5.96794	6.198963	6.144148	0.830114	6.015596	0.888223	0.82182	3.483245	3.548295	1.179724	3.112714	0.58281
ENSMUSG00000064365	mt-Ts2	4.450724	4.758022	4.913534	4.260566	4.439895	4.368212	0.475656	5.413932	4.944624	0.650229	0.502835	5.304682
ENSMUSG00000064220	Hist2h2aa1	3.137326	3.920005	3.755308	3.545681	3.681322	3.984228	0	4.947828	0	0	4.59402	4.83802
ENSMUSG00000029711	Epo	3.064989	3.084778	3.132658	0	2.949304	4.204238	2.367764	2.111797	2.973611	0	2.110641	0
ENSMUSG00000037872	Ackr1	4.631	4.55237	4.666553	4.50068	1.406237	4.428772	4.394807	4.31447	4.217929	1.424665	1.460045	2.504411
ENSMUSG00000104953	Gm47308	0.303113	0.308929	0.255961	6.125226	0.287037	6.212159	0.284458	0.321265	0.32725	5.011327	0.282934	0.304265
ENSMUSG00000086503	Xist	0.061271	3.897009	0.016739	4.193859	0.024478	0.022217	0.016205	0.00701	0.03209	0.118198	0.015815	0.030168
ENSMUSG00000098743	Gm27927	0	4.327691	0	4.652368	0	0	0	0	0	0.079767	0	0
ENSMUSG00000074506	Gm10705	4.214571	5.220398	1.965333	4.615738	3.408148	3.801138	1.589732	1.159605	3.044287	5.324437	4.672381	3.056219

### GO and KEGG Analysis for Differential Expression Genes (DEGs) and Mitochondria-Related Genes

**Figure 3:**
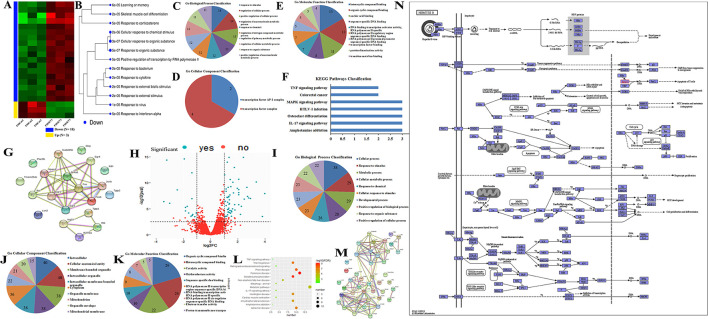
GO/KEGG analysis for differential expression genes (DEGs) and mitochondria-related genes. **(A,B)** Cluster analysis of DEGs for heatmap and dendrogram; **(C–F)** GO terms and KEGG pathways analysis of DEGs; **(G)** PPI network construction for DEGs; **(H)** the volcano plot for displaying the distribution of DEGs; **(I–K)** GO function analysis for gene sets including DEGs and mitochondria-related genes; **(L)** bubble plot showed the signal pathways of gene sets; **(M)** PPI network construction for gene sets; **(N)** presentation for involving in signal pathway.

The heatmap showed that the DEGs comprised 3 upregulated and 18 downregulated genes (Figure 3A), and these genes were enriched in various signal pathways consisting of learning or memory (Figure 3B). Then, these hub genes were further analyzed through String online tools. Briefly, the GO function analysis as a dynamic controlled vocabulary is utilized to describe the role of the gene with three categories of information comprising of biological process (BP), cellular component (CC), and molecular function (MF). GO term enrichment analysis indicated that the BP was involved in 88 categories, and the top 10 BP is presented in Figure 3C; the CC consists of 2 categories as shown in Figure 3D; and the MF includes 17 categories, and the top 10 MF is shown in Figure 3E. Meanwhile, the PPI network analysis was constructed for DEGs, the results are shown in Figure 3G, and the enriched pathways are shown in Figure 3F. The volcano plot showed the distribution of DEGs (Figure 3H). Previous studies indicated that mitochondria took part in neurogenesis and maintaining cognitive function (32, 33). Therefore, we speculated that mitochondria may involve in the sevoflurane-induced neurotoxicity. GO/KEGG analysis was performed for DEGs and mitochondria-related genes. The GO function analysis including BP, CC, and MF is shown in Figures 3I–K, respectively. The PPI network is shown in Figure 3M. The bubble diagram displayed the enriched pathways (Figure 3L), and mitochondria were involved in the signaling pathways (Figure 3N). The bioinformatic analysis results indicated that DEGs may participate in mediating cognitive deficits, which were closely correlated with the mitochondrial function.

### Sevoflurane Administration Results in Mitochondrial Dysfunction

**Figure 4:**
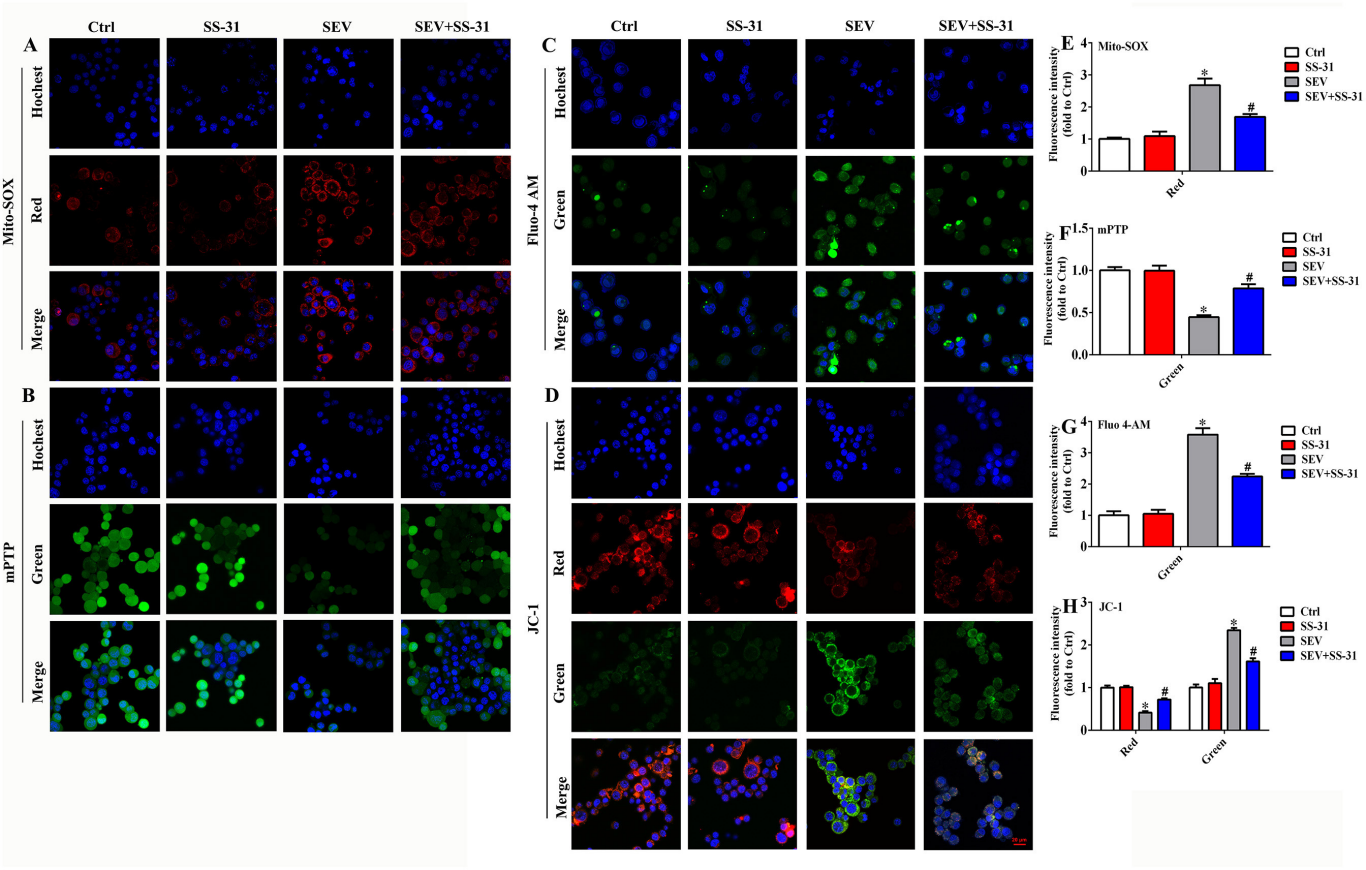
Sevoflurane administration induced mitochondrial dysfunction. **(A,E)** MitoSOX staining for detecting the level of mitochondrial ROS [one-way ANOVA, Tukey's multiple comparisons test, Treatment: *F*_(1.969, 3.938)_ = 122.4, mean difference (95% CI): −1.680 (−2.466 to −0.8936) (Ctrl vs. SEV) and 0.9879 (0.3192 to 1.657) (SEV vs. SEV+SS-31), *p* < 0.001]; **(B,F)** mitochondrial permeability transition pore assay for assessing membrane permeability [one-way ANOVA, Tukey's multiple comparisons test, Treatment: *F*_(1.612, 3.224)_ = 26.41, 0.5541 (0.3417 to 0.7666) (Ctrl vs. SEV) and −0.3411 (−0.7706 to 0.08832) (SEV vs. SEV+SS-31), *p* < 0.05]; **(C,G)** Fluo calcium indicators for investigating cytoplasm calcium levels [one-way ANOVA, Tukey's multiple comparisons test, Treatment: *F*_(1.164, 2.329)_ = 69.84, −2.578 (−3.406 to −1.749) (Ctrl vs. SEV) and 1.336 (0.06602 to 2.606) (SEV vs. SEV+SS-31), *p* < 0.01]; **(D,H)** JC-1 probe for determining mitochondrial membrane potential [red group: one-way ANOVA, Tukey's multiple comparisons test, Treatment: *F*_(1.336, 2.672)_ = 44.77, mean difference (95% CI): 0.5858 (0.4135 to 0.7580) (Ctrl vs. SEV) and −0.3051 (−0.4774 to −0.1329) (SEV vs. SEV+SS-31), *p* < 0.01]; [green group: one-way ANOVA, Tukey's multiple comparisons test, Treatment: *F*_(1.036, 2.073)_ = 65.69, mean difference (95% CI): −1.343 (−1.695 to −0.9909) (Ctrl vs. SEV) and 0.7293 (0.3774 to 1.081) (SEV vs. SEV+SS-31), *p* < 0.05]. *p < 0.05, compared with Ctrl group; ^#^*p* < 0.05, compared with SEV group.

Mitochondria are the cellular structures responsible for energy metabolism, which mainly correlated with ROS production, MMP, and cytoplasm calcium levels. Therefore, MitoSOX staining, mitochondrial permeability transition pore (mPTP) assay, Fluo calcium indicators, and JC-1 probe were used to detect the effect of sevoflurane administration on mitochondrial function, respectively. The MitoSOX staining suggested that sevoflurane administration obviously elevated the mitochondrial ROS level, whereas the supplement of SS-31 effectively reversed the ROS production in mitochondria (Figures 4A,E; *p* < 0.05). The mPTP assay results showed that the green fluorescence was reduced in the SEV group than in the Ctrl group, whereas, it was enhanced in the SEV+SS-31 group compared with the SEV group (Figures 4B,F; *p* < 0.05). Meanwhile, Fluo 4-AM fluorescence indicated that the cytoplasm calcium level was upregulated in the SEV group compared with the Ctrl group, whereas it was downregulated in the SEV+SS-31 group compared with the SEV group (Figures 4C,G; *p* < 0.05). Moreover, the mitochondrial MMP results displayed that the red fluorescence was decreased and green fluorescence was increased in the SEV group compared with the Ctrl group (Figures 4D,H; *p* < 0.05), whereas the red fluorescence was increased and green fluorescence was decreased in the SEV+SS-31 group compared with the SEV group (Figures 4D,H; *p* < 0.05). These results suggested that sevoflurane administration induced mitochondrial dysfunction, whereas the supplement of SS-31 effectively reversed this status.

### Mitochondrial TCA Cycle and Electron Transport Chain (ETC) Participate in Sevoflurane-Induced Neurotoxicity

**Figure 5:**
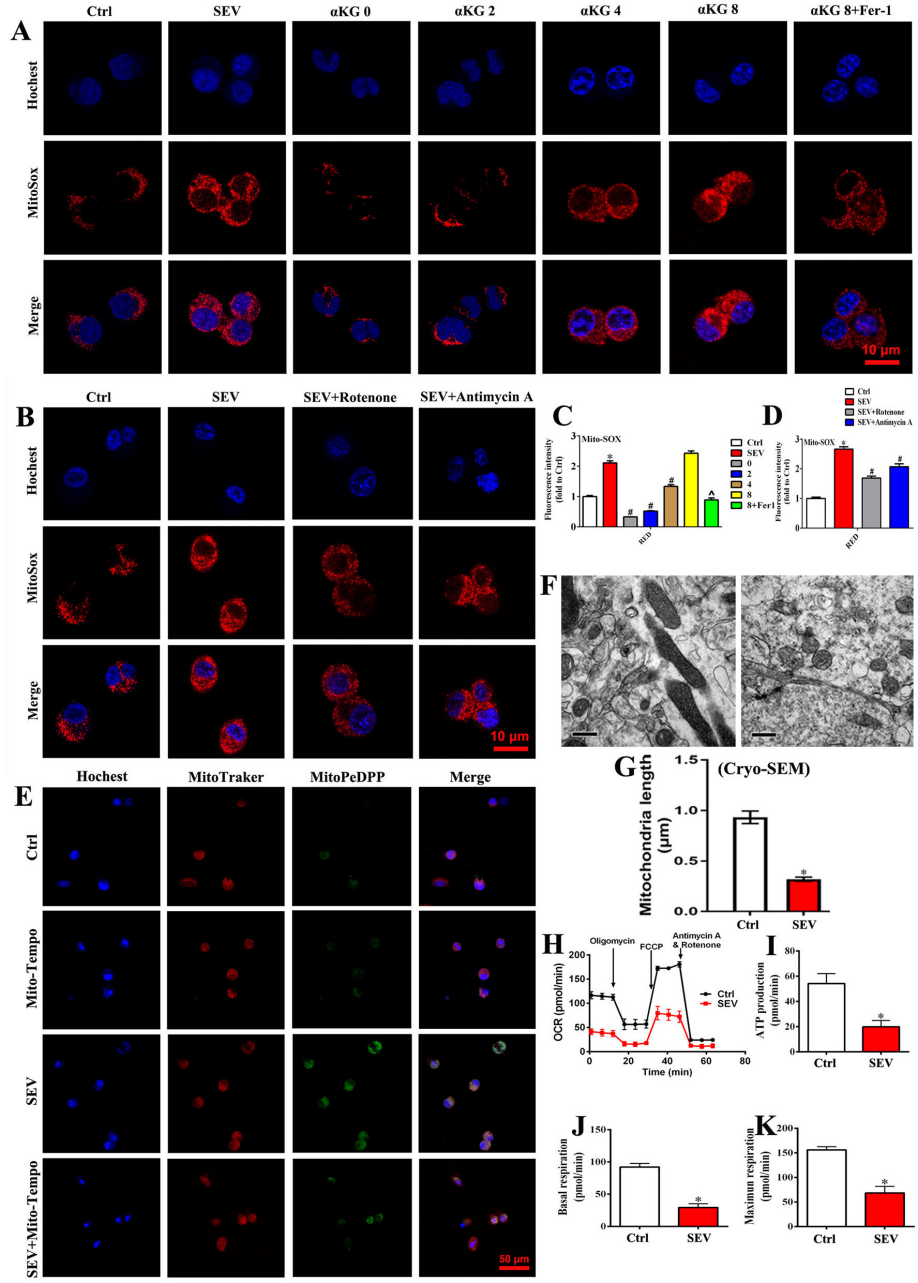
Mitochondrial TCA cycle and ETC were implicated in sevoflurane-induced neurotoxicity. **(A,C)** MitoSOX staining for investigating the role of mitochondrial TCA cycle in ROS production [one-way ANOVA, Tukey's multiple comparisons test, Treatment: *F*_(1.601, 3.201)_ = 224.9, mean difference (95% CI): −1.106 (−1.983 to −0.2299) (Ctrl vs. SEV), 1.781 (1.227 to 2.335) (SEV vs. 0), 1.587 (0.8721 to 2.302) (SEV vs. 2), 0.7720 (0.4046 to 1.139) (SEV vs. 4), −0.3208 (−1.343 to 0.7018) (SEV vs. 8) and 1.217 (0.04885 to 2.385) (SEV vs. 8+Fer1), *p* < 0.001]; **(B,D)** MitoSOX staining for determining the effect of mitochondrial ETC in ROS accumulation [one-way ANOVA, Tukey's multiple comparisons test, Treatment: *F*_(1.584, 3.671)_ = 128.4, mean difference (95% CI): −1.662 (−2.362 to −0.9620) (Ctrl vs. SEV), 0.9727 (0.5341 to 1.411) (SEV vs. SEV+Rotenone) and 0.5929 (0.4474 to 0.7384) (SEV vs. SEV+Antimycin A), *p* < 0.001]; **(E)** representative fluorescent images of mitochondrial lipid peroxidation detected by MitoPeDPP (green); **(F,G)** the mitochondrial ultrastructure in the hippocampus was observed by an electron microscope (*t-*test, *p* < 0.05); **(H–K)** detection for mitochondrial respiratory function by OCR assay (*t-*test, *p* < 0.05). Scar bar = 10 μm, applied in **(A,B)**; Scar bar = 50 μm in **(E)**. *p < 0.05, compared with the Ctrl group; ^#^*p* < 0.05, compared with the SEV group; ^∧^*p* < 0.05, compared with αKG 8 group.

To investigate the role of mitochondrial TCA cycle and ETC in sevoflurane-induced neurotoxicity, culture medium without glutamine, supplied with α-KG, rotenone, or antimycin A was used, respectively. In the absence of glutamine, mitochondrial ROS production was obviously reduced when compared to the SEV group, but the mitochondrial ROS accumulation was gradually elevated accompanied by αKG supplement (Figures 5A,C; *p* < 0.05). Meanwhile, the mitochondrial ROS level was decreased in αKG 8 group after adding the Fer-1 reagent (Figures 5A,C; *p* < 0.05). Similarly, the results showed that ETC inhibitors including mitochondrial complex I (rotenone) and complex III (antimycin A) effectively suppressed mitochondrial ROS accumulation induced by sevoflurane administration (Figures 5B,D; *p* < 0.05). Meanwhile, the oxidative lipid in mitochondria caused by sevoflurane administration was inhibited effectively by Mito-Tempo (Figure 5E). In addition, mitochondria presented decreased volume and intercristal space, and the longest diameter was reduced in the SEV group compared with the Ctrl group (Figures 5F,G). Furthermore, the OCR was performed to investigate the mitochondrial respiratory function. We found that sevoflurane administration significantly suppressed mitochondrial respiration consisting of reducing ATP production, basal respiration, and maximum respiration (Figures 5H–K, *p* < 0.05). The results indicated that mitochondrial TCA cycle and ETC were implicated in sevoflurane-induced mitochondrial dysfunction, and ROS production could be effectively scavenged by Mito-Tempo.

### Elamipretide (SS-31) Improved Behavioral Performance After Sevoflurane Exposure

**Figure 6:**
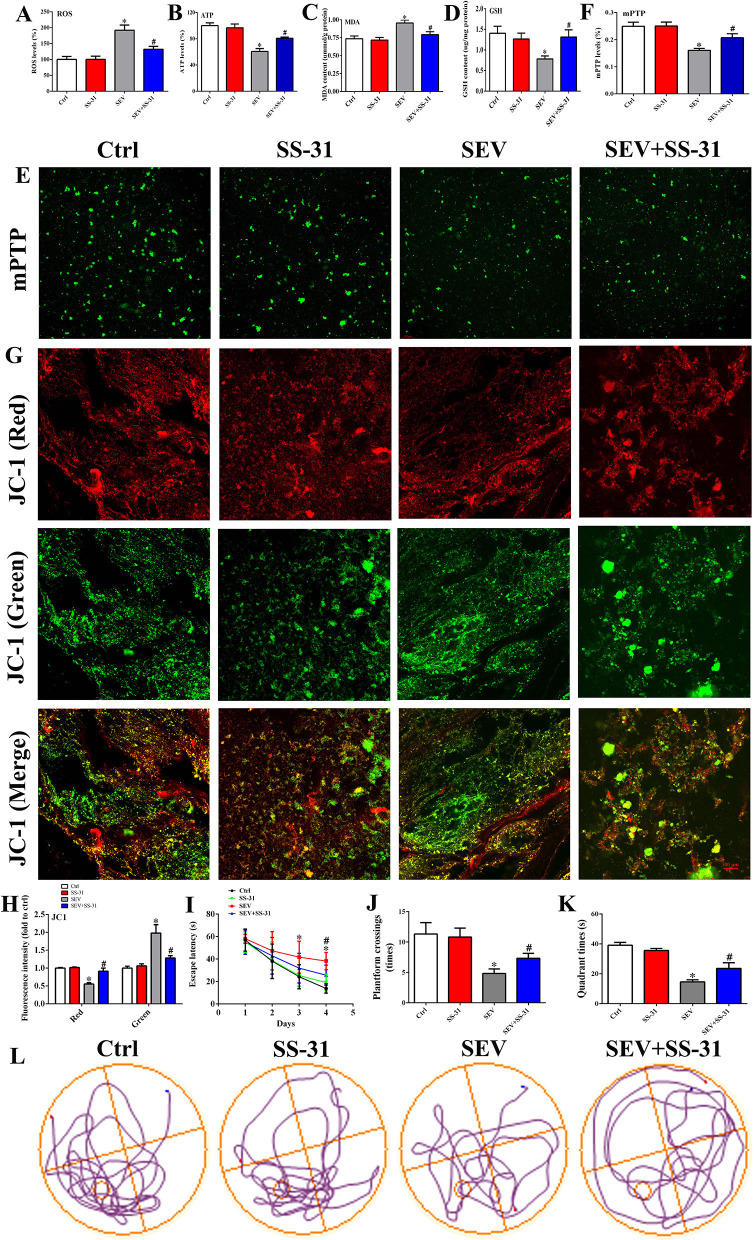
SS-31 treatment alleviated sevoflurane-induced cognitive deficits by enhancing mitochondrial function. **(A)** Detection for ROS accumulation in hippocampus [one-way ANOVA, Tukey's multiple comparisons test, Treatment: *F*_(3,20)_ = 13.68, mean difference (95% CI): −91.74 (−138.0 to −45.49) (Ctrl vs. SEV) and 59.80 (13.55 to 106.1) (SEV vs. SEV+SS-31), *p* < 0.001]; **(B–D)** measurement for ATP, MDA, and GSH level [**(B)**: one-way ANOVA, Tukey's multiple comparisons test, Treatment: *F*_(3,20)_ = 17.85, mean difference (95% CI): 39.34 (22.45 to 56.23) (Ctrl vs. SEV) and −20.02 (−36.91 to −3.127) (SEV vs. SEV+SS-31), *p* < 0.001]; [**(C)**: one-way ANOVA, Tukey's multiple comparisons test, Treatment: *F*_(3,20)_ = 7.739, mean difference (95% CI): −0.2195 (−0.3738 to −0.06522) (Ctrl vs. SEV) and 0.1625 (0.008221 to 0.3168) (SEV vs. SEV+SS-31), *p* < 0.01]; [**(D)**: one-way ANOVA, Tukey's multiple comparisons test, Treatment: *F*_(3,20)_ = 3.601, mean difference (95% CI): 0.6220 (0.1204 to 1.124) (Ctrl vs. SEV) and −0.5295 (−1.031 to −0.02790) (SEV vs. SEV+SS-31), *p* < 0.05]; **(E,F)** mitochondrial membrane permeability was evaluated by mPTP assay [one-way ANOVA, Tukey's multiple comparisons test, Treatment: *F*_(3,20)_ = 10.53, mean difference (95% CI): 0.08883 (0.04397 to 0.1337) (Ctrl vs. SEV) and −0.04683 (−0.09170 to −0.001969) (SEV vs. SEV+SS-31), *p* < 0.001]; **(G,H)** mitochondrial membrane potential was determined by JC-1 probe [Red: One-way ANOVA, Dunn's multiple comparisons test, mean difference (95% CI): 0.4482 (0.2487 to 0.6477) (Ctrl vs. SEV) and −0.3570 (−0.5565 to −0.1575) (SEV vs. SEV+SS-31), *p* < 0.001; Green: one-way ANOVA, Dunn's multiple comparisons test, mean difference (95% CI): −0.9780 (−1.485 to −0.4707) (Ctrl vs. SEV) and 0.6982 (0.1909 to 1.206) (SEV vs. SEV+SS-31), *p* < 0.01]; **(I–L)** Behavioral test by MWM assay including escape latency, target quadrant time, platform crossing number, and motion trail [**(I)**: two-way ANOVA, Bonferroni's multiple comparisons test, interaction: *F*_(9,180)_ = 2.610, mean difference (95% CI): −12.29 (−17.63 to −6.949) (Ctrl vs. SEV) and 6.222 (0.8833 to 11.56) (SEV vs. SEV+SS-31), *p* < 0.01; **(J)**: one-way ANOVA, Tukey's multiple comparisons test, Treatment: *F*_(3,20)_ = 32.91, mean difference (95% CI): 6.500 (4.383 to 8.617) (Ctrl vs. SEV) and −2.500 (−4.617 to −0.3827) (SEV vs. SEV+SS-31), *p* < 0.001; **(K)**: one-way ANOVA, Tukey's multiple comparisons test, Treatment: *F*_(3,20)_ = 138.4, mean difference (95% CI): 24.52 (20.73 to 28.30) (Ctrl vs. SEV) and −9.067 (−12.85 to −5.279) (SEV vs. SEV+SS-31), *p* < 0.001; *n* = 8]. Scar bar = 100 μm. *p < 0.05, compared with the Ctrl group; ^#^*p* < 0.05, compared with the SEV group.

To determine the role of mitochondria in sevoflurane-induced cognitive deficits, SS-31 was administrated and relevant parameters were examined. First, the hippocampal specimens were harvested, and then, the level of ROS, ATP, MDA, and GSH was detected, respectively. The results showed that the ROS level of the hippocampus was increased following sevoflurane inhalation, whereas SS-31 injection protected from oxidative stress damage (Figure 6A, *p* < 0.05). Meanwhile, the level of ATP and GSH was reduced, and the MDA content was increased in the SEV group, whereas SS-31 administration partly reversed this situation (Figures 6B–D, *p* < 0.05). Then, purified mitochondria were extracted, and the membrane permeability and MMP were detected by mPTP assay and JC-1 probe. The mPTP assay suggested that the green fluorescence was decreased in the SEV group than in the Ctrl group, whereas, it was increased in the SEV+SS-31 group compared to the SEV group (Figures 6E,F; *p* < 0.05). The mitochondrial MMP results showed that the red fluorescence intensity was reduced and green fluorescence was increased in the SEV group compared with the Ctrl group (Figures 6G,H; *p* < 0.05). Conversely, SS-31 reversed the effects of sevoflurane on MMP (Figures 6G,H; *p* < 0.05). Additionally, the behavioral performance was measured by MWM tests including escape latency, target quadrant time, platform crossing number, and motion trail. We found that sevoflurane exposure resulted in cognitive damage, and SS-31 administration significantly alleviated the behavioral deficits (Figures 6I–L, *p* < 0.05). These results indicated that SS-31 treatment attenuated sevoflurane-induced cognitive dysfunction by enhancing mitochondrial function.

### Sevoflurane Exposure-Induced Mitochondrial Dysfunction Associated With Ferroptosis

**Figure 7:**
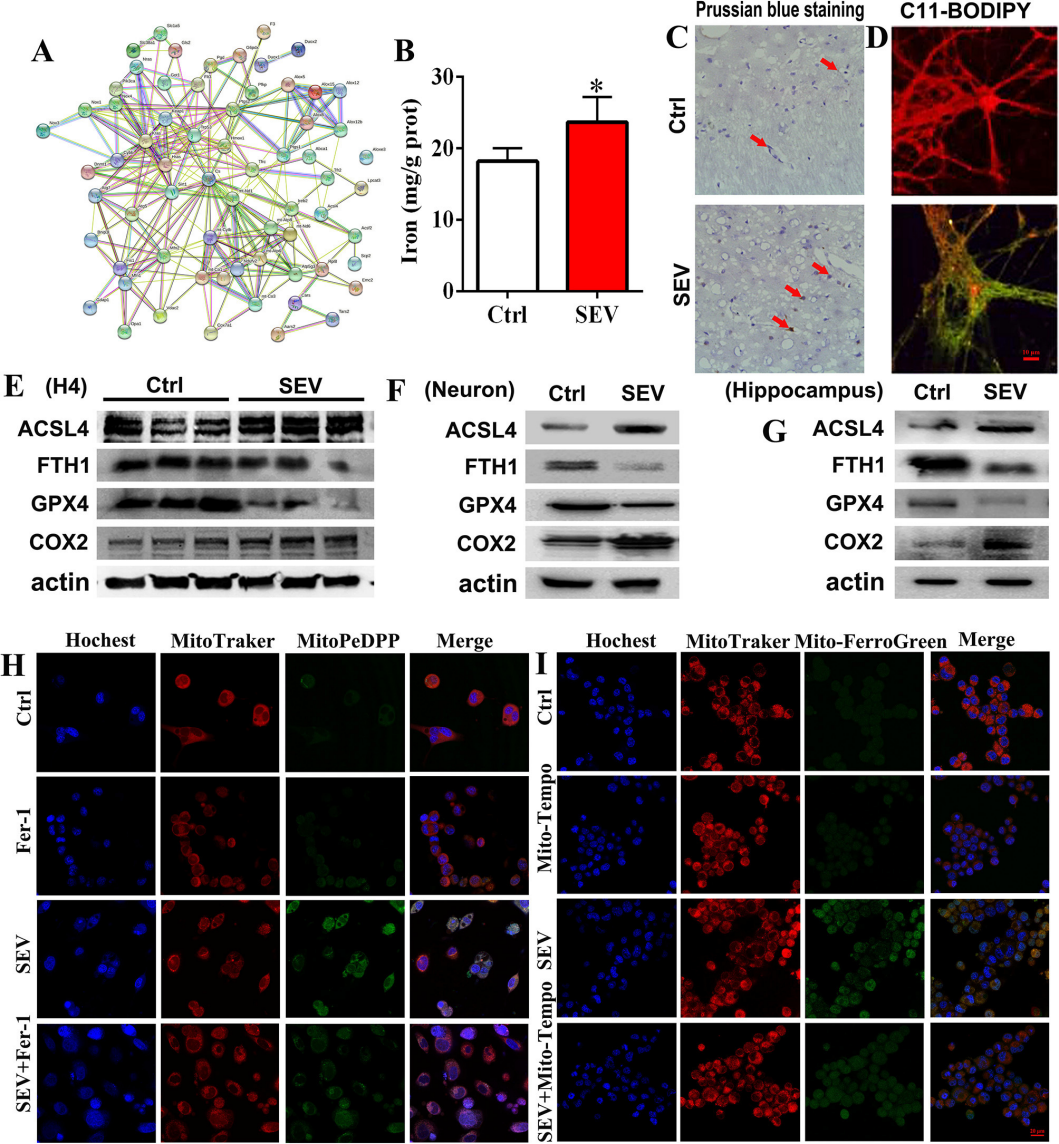
Sevoflurane-induced mitochondrial dysfunction associated with ferroptosis. **(A)** Bioinformatic analysis for mitochondria and ferroptosis-related genes; **(B,C)** detection for iron content and distribution by iron assay and Prussian blue staining (*t-*test, *p* = 0.0157); **(D)** C11-BODIPY staining for evaluating the lipid peroxidation level; **(E–G)** WB assay for measuring ACSL4, FTH1„GPX4 and COX2 protein expression; **(H)** mitochondrial lipid hydroperoxide evaluation by MitoPeDPP fluorescence dye; **(I)** mitochondrial iron content detection by Mito-FerroGreen staining. Scar bar = 10 μm, applied in **(D)**; Scar bar = 20 μm in **(H,I)**. *p < 0.05, Ctrl vs. SEV.

To determine the role of mitochondria dysfunction in ferroptosis, bioinformatic analysis and a series of molecular experiments were performed. Bioinformatic analysis suggested that there were complex regulatory relations between mitochondria and ferroptosis-related genes (Figure 7A). The iron assay showed that the hippocampal iron content was increased after sevoflurane exposure (Figure 7B, *p* < 0.05). Prussian blue staining displayed that the iron deposition of the hippocampus was apparently elevated in the SEV group than in the Ctrl group (Figure 7C). Then, C11-BODIPY dye was used to monitor the lipid peroxidation level, and we found that the fluorescence partly shifted from red to green after sevoflurane administration (Figure 7D). The WB assay showed that the protein level of ACSL4 and COX2 was upregulated, but the GPX4 and FTH1 protein expression was downregulated in the SEV group compared with the Ctrl group (Figures 7E–G). Additionally, mitochondrial lipid hydroperoxide production was increased after sevoflurane administration, whereas Fer-1 treatment reduced lipid hydroperoxide formation (Figure 7H). Sevoflurane exposure-induced excessive mitochondrial iron content was indicated by Mito-FerroGreen signals, whereas Mito-Tempo treatment reversed iron accumulation (Figure 7I). The results suggested that sevoflurane-induced mitochondrial dysfunction elicited ferroptosis.

### Chelating Neurotoxic Iron Ameliorated Cognitive Deficits After Sevoflurane Exposure

**Figure 8:**
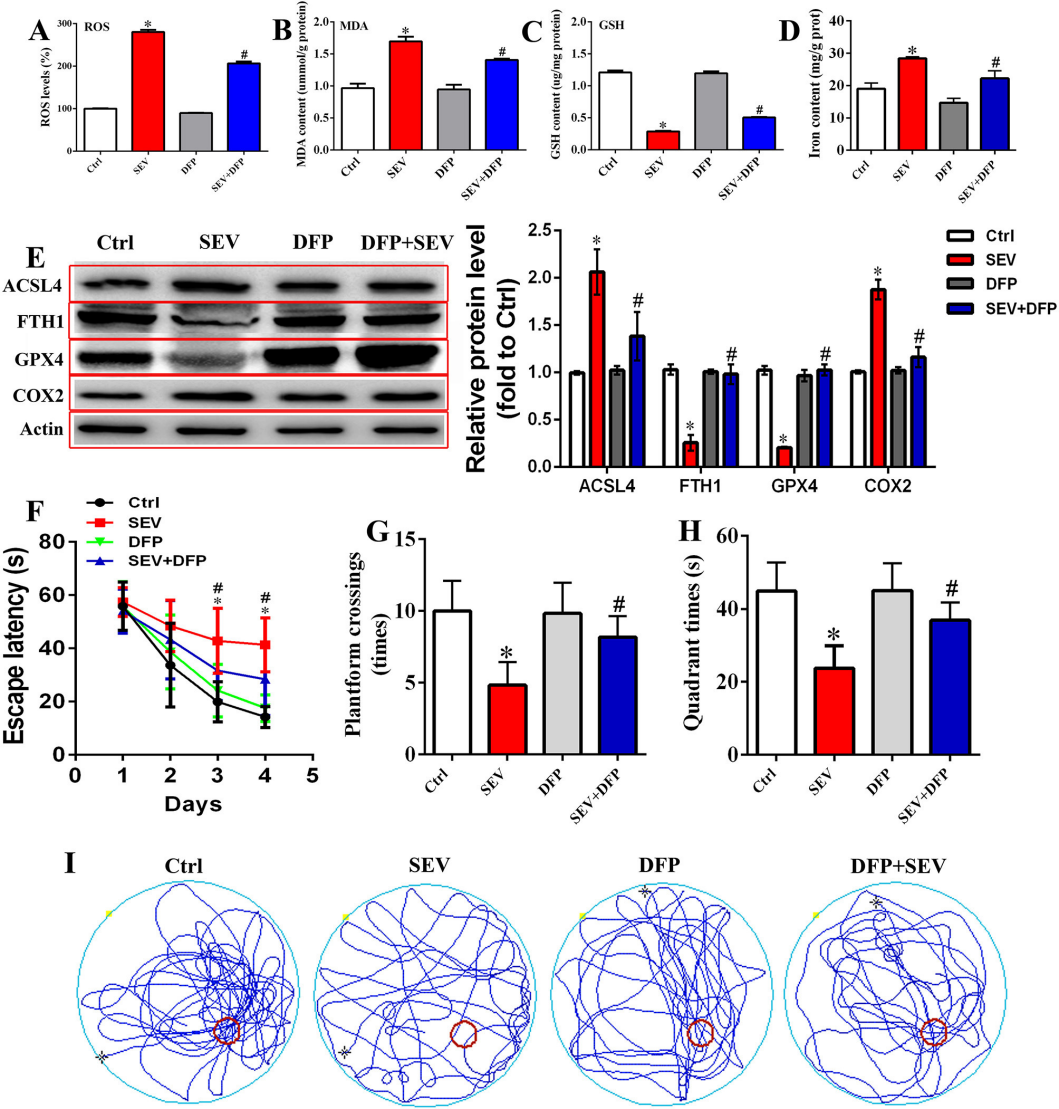
Restriction of neurotoxic iron accumulation attenuated sevoflurane-induced cognitive dysfunction. **(A)** Detection for ROS level in the hippocampus [one-way ANOVA, Tukey's multiple comparisons test, Treatment: *F*_(3,20)_ = 709.2, mean difference (95% CI): −180.0 (−193.5 to −166.5) (Ctrl vs. SEV) and 73.66 (60.15 to 87.17) (SEV vs. SEV+DFP), *p* < 0.001]; **(B–D)** measurement for MDA, GSH level, and iron content [**(B)**: one-way ANOVA, Tukey's multiple comparisons test, Treatment: *F*_(3,20)_ = 32.19, mean difference (95% CI): −0.7307 (−0.9848 to −0.4766) (Ctrl vs. SEV) and 0.2897 (0.03556 to 0.5438) (SEV vs. SEV+DFP), *p* < 0.001; **(C)**: one-way ANOVA, Tukey's multiple comparisons test, Treatment: *F*_(3,20)_ = 452.7, mean difference (95% CI): 0.9220 (0.8338 to 1.010) (Ctrl vs. SEV) and −0.2172 (−0.3054 to −0.1289) (SEV vs. SEV+DFP), *p* < 0.001; **(D)**: one-way ANOVA, Tukey's multiple comparisons test, Treatment: *F*_(3,8)_ = 37.00, mean difference (95% CI): −9.387 (−13.69 to −5.082) (Ctrl vs. SEV) and 6.179 (1.874 to 10.48) (SEV vs. SEV+DFP), *p* < 0.001]; **(E)** WB assay for assessing ACSL4, FTH1, GPX4, and COX2 protein expression [ACSL4: one-way ANOVA, Tukey's multiple comparisons test, Treatment: *F*_(3,8)_ = 23.58, mean difference (95% CI): −1.067 (−1.530 to −0.6037) (Ctrl vs. SEV) and 0.6793 (0.2160 to 1.143) (SEV vs. SEV+DFP), *p* < 0.001; FTH1: one-way ANOVA, Tukey's multiple comparisons test, Treatment: *F*_(3,8)_ = 80.13, mean difference (95% CI): 0.7734 (0.5832 to 0.9636) (Ctrl vs. SEV) and −0.7261 (−0.9163 to −0.5359) (SEV vs. SEV+DFP), *p* < 0.001; GPX4: one-way ANOVA, Tukey's multiple comparisons test, Treatment: *F*_(3,8)_ = 204.9, mean difference (95% CI): 0.8170 (0.6903 to 0.9437) (Ctrl vs. SEV) and −0.8194 (−0.9461 to −0.6927) (SEV vs. SEV+DFP), *p* < 0.001; COX2: one-way ANOVA, Tukey's multiple comparisons test, Treatment: *F*_(3,8)_ = 87.72, mean difference (95% CI): −0.8695 (−1.069 to −0.6700] (Ctrl vs. SEV) and 0.7158 (0.5163 to 0.9152) (SEV vs. SEV+DFP), *p* < 0.001]; **(F–I)** Behavioral test by MWM assay including escape latency, target quadrant time, platform crossing number, and motion trail [**(F)**: two-way ANOVA, Bonferroni's multiple comparisons test, interaction: *F*_(9,180)_ = 5.048, mean difference (95% CI): −16.55 (−20.92 to −12.18) (Ctrl vs. SEV) and 8.154 (3.785 to 12.52) (SEV vs. SEV+DFP), *p* < 0.001; **(G)**: one-way ANOVA, Tukey's multiple comparisons test, Treatment: *F*_(3,20)_ = 13.57, mean difference (95% CI): 5.167 (2.176 to 8.157) (Ctrl vs. SEV) and :3.333 (:6.324 to :0.3427) (SEV vs. SEV+DFP), *p* < 0.001; **(H)**: one-way ANOVA, Tukey's multiple comparisons test, Treatment: *F*_(3,8)_ = 37.00, mean difference (95% CI): 21.22 (10.43 to 32.00) (Ctrl vs. SEV) and :13.20 (:23.99 to :2.419) (SEV vs. SEV+DFP), *p* < 0.001; *n* = 6]. *p < 0.05, compared with the Ctrl group; ^#^*p* < 0.05, compared with the SEV group.

To estimate the relationship between iron overload and cognitive outcomes, DFP was administrated after sevoflurane inhalation. Briefly, the hippocampal tissues were acquired, and the ROS, MDA, GSH, and iron content were detected, respectively. The results showed that the ROS level of the hippocampus was increased following sevoflurane exposure, whereas DFP treatment attenuated oxidative stress damage (Figure 8A, *p* < 0.05). The MDA and iron content were increased, and the level of GSH was reduced in the SEV group, whereas DFP treatment effectively reversed this status (Figures 8B–D, *p* < 0.05). Meanwhile, the WB assay showed that the protein level of ACSL4 and COX2 was upregulated, but the GPX4 and FTH1 protein expression was downregulated in the SEV group compared with the Ctrl group (Figure 8E, *p* < 0.05), whereas DFP treatment significantly reversed above-mentioned protein expression (Figure 8E, *p* < 0.05). Additionally, the behavioral deficits were observed by MWM tests comprising escape latency, target quadrant time, platform crossing number, and motion trail following sevoflurane exposure, whereas DFP administration alleviated the cognitive dysfunction (Figures 8F–I, *p* < 0.05). These results suggested that chelating neurotoxic iron could effectively enhance sevoflurane-induced cognitive deficits.

## Discussion

Cognitive dysfunction is a common complication involving learning and memory deficits, attention and information processing anomalies, and personality and social ability disorders (34, 35). Previous studies documented that anesthesia inhalation resulted in direct toxic effects in neurons by inducing the dysregulation of calcium homeostasis and neurotransmitter release, aggravating the endogenous neurodegeneration processes, and inhibiting the physiological functions of neural stem cells (36). Accumulating evidence demonstrated that sevoflurane administration induced neuroinflammation and neuronal damage, reduced the synaptic plasticity, and eventually resulted in cognitive impairment (37). In this study, neonatal animals received sevoflurane anesthesia, and then, a behavioral test and immunofluorescent staining were employed. The results showed that the behavioral performance was significantly impaired undertaking sevoflurane exposure. Immunofluorescent outcomes displayed that the immature neurons were increased and the mature neurons were reduced in the hippocampus. Golgi-Cox staining indicated that the dendritic length, density, and nodes were obviously reduced after sevoflurane administration. Bioinformatic analysis suggested that sevoflurane administration triggered a series of aberrant gene expressions implicating in the nervous system development. Meanwhile, these DEGs may participate in mediating cognitive deficits and are closely correlated with mitochondrial dysfunction. Additionally, the level of mitochondrial lipid peroxidation, MMP and membrane permeability, cytoplasm calcium levels, and iron content were investigated after sevoflurane treatment, respectively. Our results indicated that sevoflurane administration resulted in mitochondrial dysfunction, and inducing ferroptosis, whereas SS-31 and DFP treatment effectively attenuated cognitive impairment.

Mitochondria, as one of the chief sources of reactive oxygen species (ROS), play a crucial role in maintaining normal physiological activities. Excessive stimulation of NAD(P)H and electron transport chain would disrupt the normal redox state and cause the overproduction of peroxides and free radicals, thereby leading to oxidative damage and mitochondrial dysfunction (38). In this study, we found that the mitochondrial TCA cycle and ETC participated in ROS production after sevoflurane exposure, which may induce mitochondrial DNA damage. Thereafter, the mitochondrial DNA impairment derived from an accumulation of superoxide radicals would further amplify oxidative stress by mediating critical proteins and initiating a vicious circle of ROS production to destroy the organelle, induce metabolic disequilibrium and genomic instability, and eventually result in neuronal apoptosis (39–41). The hippocampus is one of the most vulnerable brain regions to oxidative damage, which is critical for the formation of long-term memory and learning (42). Of note, calcium, as a pivotal signaling ion, regulates various physiological/pathological cellular responses involved in eliciting enzyme secretion and activating apoptosis (43). Calcium overload triggers a serial cascade of pathophysiologic reactions including metabolic disequilibrium, aberrant neurotransmitter release, and disturbing synaptic transmission, which ultimately induced cognitive disorders (44). Mitochondrial uptake of calcium maintains the intracellular calcium homeostasis, whereas oxidative stress contributes to calcium overload and in turn activates oxidative metabolism pathways, thereby triggering the opening of mPTP and inducing the loss of MMP and inflammatory response (45). A related study revealed that sevoflurane contributed to intracellular calcium accumulation and thereby induced mitochondrial damage and mitochondria-mediated hippocampal neuroapoptosis (46). Consistently, we found that sevoflurane administration elevated the mitochondrial ROS level, increased the cytoplasm calcium content, reduced the mitochondrial MMP, induced the opening of mPTP, and eventually resulted in cognitive deficits, whereas SS-31 treatment effectively reversed these pathologic processes. These results provided abundant evidence for clarifying the potential mechanism regarding sevoflurane exposure-induced neurotoxicity, which implicated in destroying mitochondrial respiratory network, accelerating mitochondria ROS production, and disturbing calcium homeostasis.

Iron is a crucial component for biochemical reactions including cellular metabolism, synthesis of DNA, RNA, and proteins, enzymatic reactions, and synthesis of myelin (47). Generally, free iron could react with oxygen in metabolically active cells to generate hydroxyl radicals and hydroxyl anions, which further participate in chemical implications including lipid peroxidation, DNA strand breaks, and protein modifications and ultimately induce cell death (48). Emerging evidence revealed that neurons were particularly vulnerable to the alteration of iron content, and iron homeostasis disorder would result in significant neurotoxicity and neurogenetic abnormality, disturbing neurotransmitter synthesis and release, and mitochondrial functions (49). Previous studies suggested that hippocampal iron dyshomeostasis causes oxidative stress damage and cognitive deficits (50). Interestingly, aberrant iron deposition was observed in the hippocampus after sevoflurane inhalation. Therefore, we speculated that metabolic disturbance of iron may be involved in sevoflurane-induced neurogenetic abnormality. The developing hippocampus is susceptible to perturbations by iron content (51). Herein, we consider that sevoflurane administration induced iron dyshomeostasis resulted in neurodevelopmental disorders in the hippocampus. Mitochondria are the major generators of iron-sulfur clusters (ISCs) and tightly regulated iron uptake and utilization (52). Related research proved that sevoflurane disrupted iron homeostasis by affecting the protein expression and mitochondrial iron accumulation (53). The prevailing hypothesis indicated that the mitochondrial iron content was affected by the labile iron pool in the cytosol, and iron was transported into the mitochondria by binding with hydrophobic pockets of chaperone proteins (54). Consequently, we hypothesized that mitochondria-mediated iron metabolism may play a crucial role in sevoflurane-induced cognitive dysfunction. In this study, the WB assay showed that the protein level of ACSL4, COX2, ferroportin 1, and DMT1 was upregulated, and the GPX4 and FTH1 protein levels were downregulated after sevoflurane administration, whereas DFP treatment reversed these proteins expression, reduced ROS production, and enhanced the behavioral performance after sevoflurane exposure. Therefore, the data indicated that sevoflurane administration induced mitochondrial dysfunction, then disturbed iron homeostasis, and exacerbated neurodevelopmental disorders, while chelating neurotoxic iron effectively ameliorated behavioral malfunction.

Collectively, these findings demonstrated that oxidative stress induced by sevoflurane exposure deranged the mitochondrial respiratory chain and calcium homeostasis, disturbed the mitochondrial membrane permeability and MMP, and further initiated iron dyshomeostasis. These changes accelerated the neuronal dysfunction and eventually resulted in cognitive deficiency, whereas improving mitochondrial function and chelating neurotoxic iron effectively reversed these pathological processes.

## Data Availability Statement

The original contributions presented in the study are publicly available. This data can be found here: NCBI Sequence Read Archive (SRA) database, under accession: PRJNA781082.

## Ethics Statement

The animal study was reviewed and approved by Animal Care and Use Committee of Zhejiang University.

## Author Contributions

All authors listed have made a substantial, direct, and intellectual contribution to the work and approved it for publication.

## Funding

This research was supported by the National Natural Science Foundation of China (No. 82171176 and No. 82001424), and the Key Program of the Natural Science Foundation of Zhejiang, China (No. LZ19H090003).

## Conflict of Interest

The authors declare that the research was conducted in the absence of any commercial or financial relationships that could be construed as a potential conflict of interest.

## Publisher's Note

All claims expressed in this article are solely those of the authors and do not necessarily represent those of their affiliated organizations, or those of the publisher, the editors and the reviewers. Any product that may be evaluated in this article, or claim that may be made by its manufacturer, is not guaranteed or endorsed by the publisher.
